# Transcriptome analysis of sex-related genes in the blood clam *Tegillarca granosa*

**DOI:** 10.1371/journal.pone.0184584

**Published:** 2017-09-21

**Authors:** Heng Chen, Guoqiang Xiao, Xueliang Chai, Xingguan Lin, Jun Fang, Shuangshuang Teng

**Affiliations:** 1 Zhejiang Mariculture Research Institute, Wenzhou, Zhejiang, China; 2 Zhejiang Key Laboratory of Exploitation and Preservation of Coastal Bio-resource, Wenzhou, Zhejiang, China; 3 Engineering Research Center for Marine Bivalves, Chinese Academy of Fishery Sciences, Wenzhou, Zhejiang, China; Zhejiang University College of Life Sciences, CHINA

## Abstract

**Background:**

Blood clams (*Tegillarca granosa*) are one of the most commercial shellfish in China and South Asia with wide distribution in Indo-Pacific tropical to temperate estuaries. However, recent data indicate a decline in the germplasm of this species. Furthermore, the molecular mechanisms underpinning reproductive regulation remain unclear and information regarding genetic diversity is limited. Understanding the reproductive biology of shellfish is important in interpreting their embryology development, reproduction and population structure. Transcriptome sequencing (RNA-seq) rapidly obtains genetic sequence information from almost all transcripts of a particular tissue and currently represents the most prevalent and effective method for constructing genetic expression profiles.

**Results:**

Non-reference RNA-seq, an Illumina HiSeq2500 Solexa system, and *de novo* assembly were used to construct a gonadal expression profile of the blood clam. A total of 63.75 Gb of clean data, with at least 89.46% of Quality30 (Q30), were generated which was then combined into 214,440 transcripts and 125,673 unigenes with a mean length of 1,122.63 and 781.30 base pairs (bp). In total, 27,325 genes were annotated by comparison with public databases. Of these, 2,140 and 2,070 differentially expressed genes (DEGs) were obtained (T05 T08 vs T01 T02 T04, T06 T07 vs T01 T02 T04; in which T01-T04 and T05-T08 represent biological replicates of individual female and male clams, respectively) and classified into two groups according to the evaluation of biological replicates. Then 35 DEGs and 5 sex-related unigenes, in other similar species, were investigated using qRT-PCR, the results of which were confirmed to data arising from RNA-seq. Among the DEGs, sex-related genes were identified, including forkhead box L2 (*Foxl2*), sex determining region Y-box (*Sox*), beta-catenin (*β-catenin*), chromobox homolog (*CBX*) and Sex-lethal (*Sxl*). In addition, 6,283 simple sequence repeats (SSRs) and 614,710 single nucleotide polymorphisms (SNPs) were identified from the RNA-seq results.

**Conclusions:**

This study provided the first complete gonadal transcriptome data for the blood clam and allowed us to search many aspects of gene sequence information, not limited to gender. This data will improve our understanding of the transcriptomics and reproductive biology of the blood clam. Furthermore, molecular markers such as SSRs and SNPs will be useful in the analysis of genetic evolution, bulked segregant analysis (BSA) and genome-wide association studies (GWAS). Our transcriptome data will therefore provide important genetic information for the breeding and conservation of germplasm.

## Introduction

The blood clam (*Tegillarca granosa*) belongs to the family Arcidae, and inhabits the intertidal zone of the Indian Ocean and the western Pacific Ocean. As a shellfish with significant economic value, the blood clam has been widely cultivated across countries in Southeast Asia over the years. This species has been farmed in China for many years and is distributed from the Shandong Province to Guangxi Province. Indeed, Zhejiang Province produced over 353 kilotons of blood clam in 2014, accounting for 38% of the total national production[1]. Blood clams are able to tolerate wider ranges of temperature and saline concentrations, and exhibit significant resistance to environmental stress. Consequently, this species is easily bred on a large scale.

Over the past two decades, a reduction in blood clam germplasm resources has become increasingly evident, predominantly due to artificial inbreeding and poor environmental conditions. Collectively, these factors have resulted in reduced genetic heterozygosity, poor resistance, precocious puberty and thin shells. In addition, studies have shown a reduction in sperm and egg quality, as well as a reduction in the fecundity of females, leading to a rapid decline in the number of offspring. Lastly, existing studies have only addressed the medicinal properties of blood clams, including their anti-cancer[2–4], antioxidant[5] and antibacterial[6–8] properties, while research targeting the protection of germplasm resources, such as its growth and reproduction, are scarce. Elucidating the reproductive biology of shellfish is important in understanding the embryonic and individual development, reproduction and population structure of this important commercial resource.

The process of sex determination regulates the differentiation of the original gonad into either testis or ovaries, and includes genetic sex determination (GSD) and environmental sex determination (ESD). Sex differentiation is the evolutionary process that causes genetic sex to create a series of gender characteristics. However, this process is controledmulti-factorial and involves sex chromosomes, chromosome ploidy, as well as other genetic factors. Furthermore, environmental factors, such as light exposure, temperature, nutritional conditions, and reproductive endocrinology can cause sex differentiation to deviate from original intent [9]. Moreover, genetic effects of sex determination have become increasingly obvious over evolutionary periods. Bivalves belonging to primitive species and show extraordinary diversification in terms of sex determination mechanisms, which vary among phylogenetically closely-related species, as well as within a single species[9–10]. Consequently, it is vital for blood clams to build up their own transcriptome, thus laying the foundation for further research in this important species.

All genetic and environmental factors are controlled by sex-related genes in order to regulate sexual development and sex differentiation. There have been many studies carried out examining the sex-related genes of vertebrates. In mammals, sex determining region Y (*Sry*)[11] and anti-Mullerian hormone (*AMH*)[11–13] are the two predominant promoters required for testis determination, and the sex determining region Y-box (*Sox9*)[14–15] is the only target identified for *Sry*. A number of genes have also been involved in the regulation of *Sry* and *AMH* expression, for example, steroidogenic factor 1 (*Sf1*)[16], *GATA4*[17–18], Wilms tumor 1 (*WT1*)[17,19] and Lim homeobox protein 9 (*Lhx9*)[20]. Indeed, research on a range of species from worms to mammals has shown that doublesex- and Mab-3-related transcription factors (*DMRT*) are the most well conserved male sex-determining genes[21]. The DMRT family shares a DM domain, which was first identified in *Drosophila melanogaster* (doublesex; *dsx*) and *Caenorhabditis elegans* (*mab-3*)[22–23] and later in *Oryzias latipes* (Y-linked DM domainGene: *DMY*)[24], *Xenopuslaevis* (W-linked DM domain gene: *DMW*, ovary-determinating)[25] and *DMRT1* in fish, birds and mice[24,26,27]. Compared to testis-related genes, however, genes expressed by the ovary have been studied far less extensively. The most important pathway of ovary development is the Wnt/beta-catenin signaling pathway, including Wnt family member 4 (*Wnt4*)[28], beta-catenin (*β-catenin*)[29] and R-spondin1 (*Rspo1*)[30]. The roles of forkhead box L2 (*Foxl2*)[31] and the dosage-sensitive sex reversal-adrenal hypoplasia congenital (*AHC*) critical region on the X chromosomegene 1 (*Dax1*)[32] has also been extensively studied during ovarian development.

Since the discovery of *Dsx* and *Mab* in invertebrates, many other sex-related genes have been identified, such as fruitless (*Fru*)[33], sex-lethal (*Sxl*), transformer (*Tra*, *Tra-2*)[34] in *D*. *melanogaster* and XO lethal (*Xol*)[35], sex-determination and dosage compensation defect (*Sdc*)[36], hermaphroditization (*Her*)[37], *Tra*[38] and feminization (*Fem*)[39–40] in *C*. *elegans*. In *D*. *melanogaster*, *Sxl* expression in females is activated in a dose-dependent manner by the X chromosome (X:A = 1.0); the activated *Sxl* transcripts encode functional proteins which cannot be produced in males. *Sxl* proteins subsequently splice their own transcripts as well as those of *Tra*, and *Tra* functions in cooperation with *Tra-2* to alternatively splice *Dsx*^F^ and *Fru*^F^ transcripts[34]. Finally, downstream genes encode transcription factors which promote female-specific development. In males, a single dose of X chromosome blocks this cascade of splice regulation, and as a result, *Dsx*^M^ is produced which causes the development of a male-specific pathway [34, 41]. In *C*. *elegans*, a double dose of X chromosome represses the activity of *Xol-1*, stimulating the expression of *Sdc*, which represents a classical pathway of X chromosome dosage compensation. In the female (hermaphroditic) sex-determination pathway, *Sdc* inhibits *Her-1* to upregulate *Tra-2* which is repressed by *Her-1* protein. *Fem* forms a complex with Cullin-2-like ubiquitinligase (*Cul-2*), which targets *Tra-1A* for proteasome-mediated degradation. However, *Fem* is downregulated by *Tra-2*, and *Tra-1A* represses *mab-3*, leading to the transcription of hermaphroditic genes. In males, *Fem* can combine with *Cul-2* and degrade the target *Tra-1A*, leading to the activation of male-specific genes[36, 38]. Nevertheless, studies involving non-model invertebrates are rare, especially in bivalves[42–45].

RNA-seq is a technique arising from high-throughput sequencing and is commonly used for the analysis of differentially expressed genes (DEGs), functional gene mining, and transcriptional profile construction. This method has proven high throughput, low cost, high accuracy, and rapid processing time [46]. Research involving RNA-seq has focused mainly upon developmental regulation, environmental stress, and biotic stress. In recent years, a range of molluscs have been used in gonadal transcriptome studies[47–51]. However, unlike other families of bivalves, which have doubly uniparental inheritance (DUI) and sex reversal[52–55], the blood clam is a hermaphroditic shellfish, exhibiting 38 diploid chromosomes without sex chromosomes or sex reversal[56]. In the blood clam, sex is more likely to be dominated by the interaction of multiple genes.

In the present study, the transcriptomes of four mature males and females were sequenced in order to search for DEGs which could be used in subsequent research involving protein structure prediction and functional analysis. Such data could be used to identify the specific pathways of sex determination and establish a suitable foundation for population genetic breeding.

## Materials and methods

### Ethics statement

The blood clam is a new breed of shellfish referred to as ‘No. 1 Yueqing Bay’cultivated by Zhejiang Mariculture Research Institute. Samples were collected from Wenling (28°17’7.83”N, 121°14’25.56”E, Taizhou, China) on June 10^th^ 2015 for scientific purposes. The shellfish were starved for two days in Qingjiang Station (Wenzhou, China) to eliminate effects of the hepatopancreas, and the gonadal tissues were dissected, immediately immersed in liquid nitrogen, packed with dry ice and sent to Biomarker Technologies Corporation for RNA-seq.

### Sample collection and RNA extraction

The blood clams collected were mature and approximately two years old. Their shells had a mean length of 30.62±2.28 mm, mean height of 24.33±1.93 mm, mean width of 21.07±1.56 mm and a mean weight of 9.96±2.29 g. The testes of the blood clam were filled with white sperm, while the ovaries were filled with orange eggs, which can be easily identified with the naked eye. Gonadal tissues were dissected, and total RNA was isolated using an EASYspin Plus Tissue and Cell RNA Rapid Extraction Kit (Aidlab, Beijing, China), which also removed genomic DNA. RNA quality was then determined with a 2100 Bioanalyser (Agilent Technologies, CA, USA) and quantified using a NanoPhotometer spectrophotometer (Thermo Fisher Scientific, Wilmington, DE).

### cDNA library preparation, Illumina sequencing and quality control

Four RNA samples from each group (males and females) were sent to Biomarker Technologies Corporation (Beijing, China) for cDNA library construction and sequencing. RNA sequencing libraries were generated using the NEBNext® Ultra RNA Library Prep Kit for Illumina (New England Biolabs, Ipswich, MA, U.S.A.) with multiplexing primers, according to the manufacturer’s protocol. cDNA libraries were constructed with average inserts of 200 base pairs (bp). In brief, mRNA was purified from total RNA using NEBNext Oligo d(T)_25_ beads, and fragmentation was carried out for first strand cDNA and second strand cDNA synthesis. After fragment purification using AMPure XP Beads (Beckman Coulter, Inc.), the short cDNA fragments were subjected to end repair and adapter ligation. Then, ligation reaction was purified for PCR library enrichment by 12–15 PCR cycling. Sequencing was performed via paired-end 25 cycles rapid run on an Illumina HiSeq2500. In addition, Q20, Q30, GC-content and sequence duplication level were calculated to assess the quality of clean data.

### De novo assembly, quality control and functional gene annotation

High-quality clean reads were obtained by removing the adaptor sequences, duplicated sequences, ambiguous reads and low-quality reads. *De novo* assembly was then accomplished using Trinity software. Clean reads were fragmented and recombined into long fragments by overlap named contigs. Related contigs were clustered using TGICL software[57] to yield unigenes that could not be extended on either end, and redundancies were removed to acquire non-redundant unigenes. We then assessed the quality of unigenes by testing the randomness of inserts, insert length and saturation measurement of the transcriptome data. Unigenes were then annotated using blastx against the Nr database (NCBI non-redundant protein sequences), KEGG (Kyoto Encyclopedia of Genes and Genomes), GO (Gene Ontology), and COG (Cluster of Orthologous Groups) to obtain protein functional annotation based upon sequence similarity. ESTScan software[58] was used to determine the sequence direction of unigenes which could not be aligned to any of the above databases. The Blast2GO program[59]was then used to retrieve GO annotations of unigenes with an E-value threshold of 1x10^5^ for further functional categorization, including cellular components, molecular functions and biological processes. Finally, WEGO software[60] was used to plot the distribution of GO functional classification of the unigenes.

### Gene expression quantification

Fragments per kilobase of transcript per million mapped reads (FPKM) was applied to evaluate gene expression levels in different samples, thus eliminating the influence of different gene lengths and sequencing levelson the calculation of gene expression.

### Correlation assessment of biological replicates

Recent studies have demonstrated that there is biological variability in the expression of genes among different individuals[61]. In order to eliminate biological variability among different individuals, four males and four females (not pooled) were investigated using the same conditions. Pearson’s correlation coefficient (r)[62] was used as an evaluation index for biological replicates; a value of r^2^ close to 1 indicated a strong correlation between the two repeated samples.

### Differentially expressed genes (DEGs)

The DESeq[63]package was used to identify DEGs and P values were corrected using the Benjamini-Hochberg method. An False Discovery Rate (FDR)<0.01 (corrected *P* values) and |log_2_FC (Fold Change)|≥1 were set as thresholds in order to identify significant DEGs between two samples. In our study, two groups (T05 T08 *versus* T01 T02 T04, T06 T07 *versus* T01 T02 T04; where T01-T04 were females and T05-T08 were males; T03 was eliminated from analysis and males were grouped into T05 + T08 and T06 + T07 according to r^2^ value) were assigned to acquire DEGs according to the correlation assessment of biological replicates. A volcano plot was created to show the significance of differential genes and a Venn diagram was created to identify similarities and differences between groups. Finally, a hierarchical cluster analysis was performed to display differential expression patterns of genes in different experimental conditions.

### Quantitative real-time PCR validation

FDR<0.001 and |log2FC|≥1 were set as thresholds for identifying positive and significant DEGs from transcriptome sequencing data. In total, 40 unigenes, consisting of 16 up-regulated ovarian unigenes and 19 down-regulated testicular unigenes and 5 sex-related unigenes in other similar species, were investigated. *18SrRNA* (F:5'-CTTTCAAATGTCTGCCCTATCAACT-3', R:5'-TCCCGTATTGTTATTTTTCGTCACT-3')[64] and *β-actin* (F:5'-GCCGCTTCTTCATCCTCAT-3', R:5'- GTCGGCAATACCTGGGAAC-3')[65] were used as reference genes. RNA templates were extracted from three mature males and three mature females and were then reverse transcribed to cDNA using the PrimeScript^TM^RT reagent kit with gDNA Eraser (TakaRa, Japan) and stored at -20°C to await qRT-PCR.

qRT-PCR was carried with a StepOnePlus^TM^real-time PCR system using SYBR^®^Green I (TakaRa, Japan) according to the manufacturer’s instructions. cDNAs were diluted five-fold for the final amplified templates of target and reference genes. Two-step qRT-PCR cycles were as follows: 95°C for 30s, followed by 40 cycles of 95°C for 5s and 60°C for 30s. The specificity of amplification was measured by melt curve analysis, which needed to be a single peak. Relative expression profiles of target genes were analyzed using the 2^-ΔΔCт Mean^method, where CT values of reference genes were calculated with a geometrical mean. Significant differences (*P*<0.05) were determined by the Student’s t-test using SPSS17.0.

### Simple sequence repeats (SSR) and single nucleotide polymorphism (SNP)

MicroSAtellite (MISA) software[66] was used to detect SSR markers, including mono-, di-, tri-, quad-, penta-, and hexa-nucleotide repeats. Tool Kit (GATK)[67] was used to select SNP loci from our transcriptome using the following parameters: a continuous single base mismatch was not ≤3 within the range of 35bp, while the quality value of SNP was >2.0 after sequence standardization.

## Results

### Evaluation of biological replicates

Biological replicates were designed to acquire more reliable DEGs and r^2^ was calculated to evaluate correlation of the samples. Based upon the r^2^ values illustrated in Table 1, two groups of males *versus* females (T05 T08 *versus* T01 T02 T04, T06 T07 *versus* T01 T02 T04) were constructed, eliminating significantly differential individuals.

**Table 1:** Correlation analysis between two selected individual blood clams.

	T01	T02	T03	T04	T05	T06	T07
T02	0.8843						
T03	0.7863	0.8641					
T04	0.8557	0.9085	0.7593				
T05	0.6415	0.7548	0.7108	0.6454			
T06	0.5396	0.4673	0.4125	0.4078	0.7390		
T07	0.5991	0.5164	0.4338	0.4442	0.7004	0.8368	
T08	0.4502	0.5410	0.5555	0.4406	0.8801	0.7267	0.6601

Values shown represent r^2^ value. T01-T04 are females and T05-T08 are males. The condition (r^2^>0.82) was used to eliminate differential individuals. T01, T02, T04 were similar, thus eliminating T03 which was significantly different. T05 is similar to T08 while T06 is similar to T07. However, T05 was not similar to T06. Therefore, we constructed two groups for analysis (T05 T08 *versus* T01 T02 T04, T06 T07 *versus* T01 T02 T04) to search for more differentially-expressed genes (DEGs).

### De novo assembly and functional gene annotation

**Fig 1:**
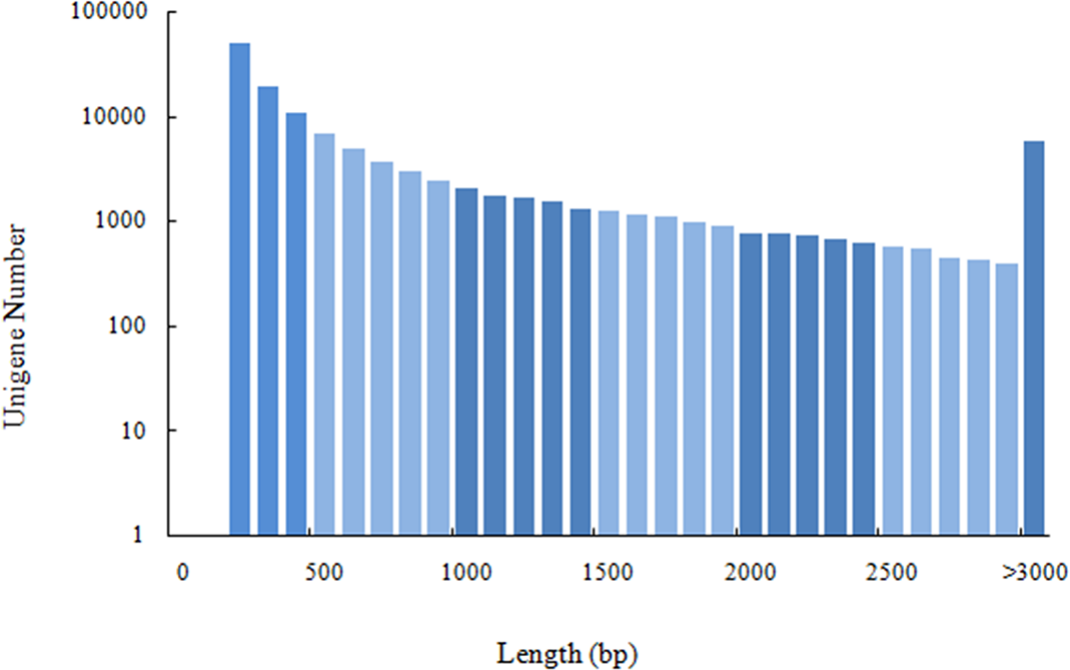
Length distribution of unigenes showing assembly quality of the blood clam transcriptome.

Eight mature blood clam gonads were sequenced, generating 63.75 Gb of clean data with at least 89.46% of Q30 from each sample (Table 2), the data had been submitted to NCBI (accession number: SRR5512703, SRR5515063). All clean data were combined into 214,440 transcripts and 125,673 unigenes with a mean length of 1,122.63 and 781.30 bp. The length distribution of unigenes is shown in Fig 1. Bioinformatic analysis of unigenes produced 27,325 annotated genes, featuring in Nr (97.7%), Swissprot (57.6%), PFAM (65.0%), KOG (53.8%), KEGG (25.1%), GO (24.3%) and COG (25.9%) databases in which there was 48% functional annotations from *Crassostrea gigas*.

**Table 2:** Quality control analysis for RNA-seq data.

Sample	ID	GC (%)	Q20 (%)	Q30 (%)
♀1	T01	37.48	94.06	89.46
♀2	T02	37.05	94.20	89.78
♀3	T03	36.86	94.22	89.77
♀4	T04	37.65	94.22	89.77
♂1	T05	36.82	94.50	90.22
♂2	T06	37.04	94.51	90.25
♂3	T07	37.29	94.28	89.84
♂4	T08	36.25	94.45	90.16

Q (Quality) -score represents the accuracy of base recognition, Q-score = -10*log P where P represents the probability of an error in base recognition (Q30>85%), and GC (%) represents the proportion of GC bases of the entire database for each individual blood clam (T01-T08).

### DEG analysis

**Fig 2:**
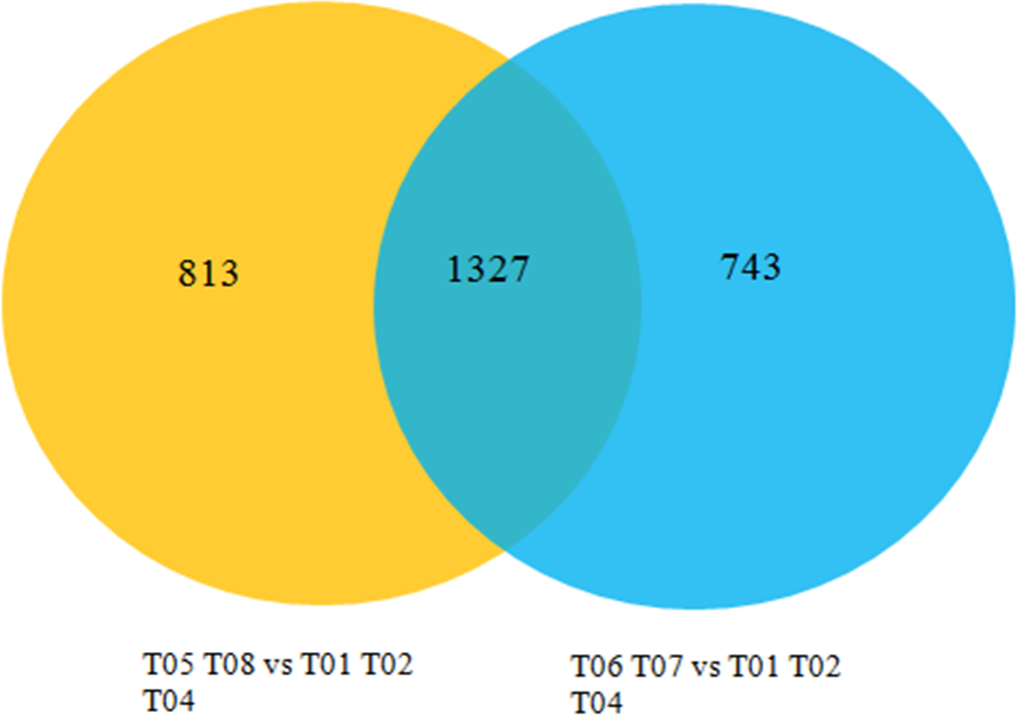
Venn diagrams for DEG relationships between two groups of blood clam showing the common differentially expressed genes (DEGs) and specific DEGs in each gene set.

**Fig 3:**
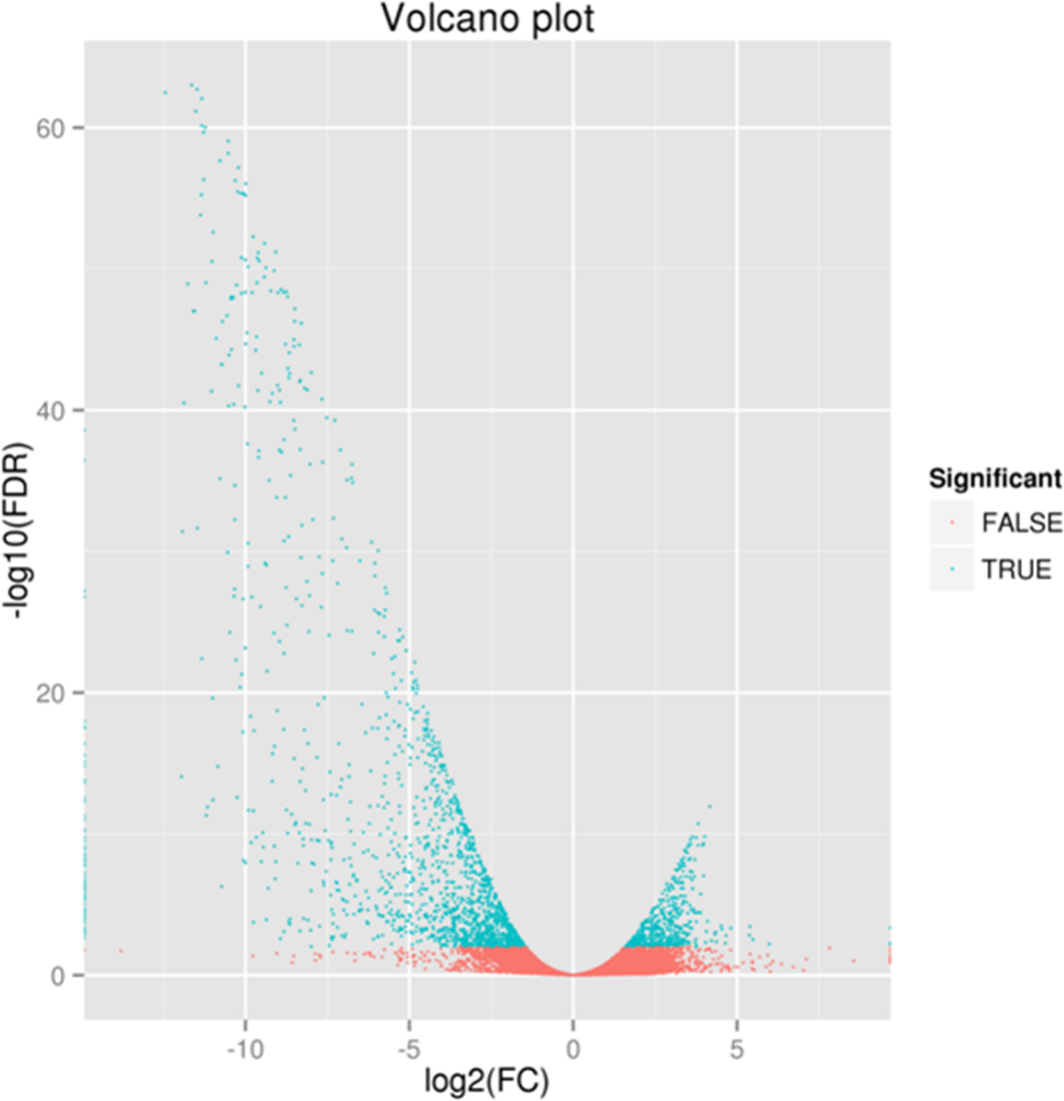
Volcano plot for group analysis of blood clams (T05 T08 *versus* T01 T02 T04). We used specific criteria to identify the significance of differential expression: an FDR (False Discovery Rate)<0.01 and |log_2_FC (Fold Change)|≥1. The left side of 0 on the x-axis represents male-biased differentially expressed genes (DEGs) while the right side of 0 represents female-biased DEGs. The green region shows significant differences (an FDR<0.01), while the red region shows non-significant differences (an FDR≥0.01).

**Fig 4:**
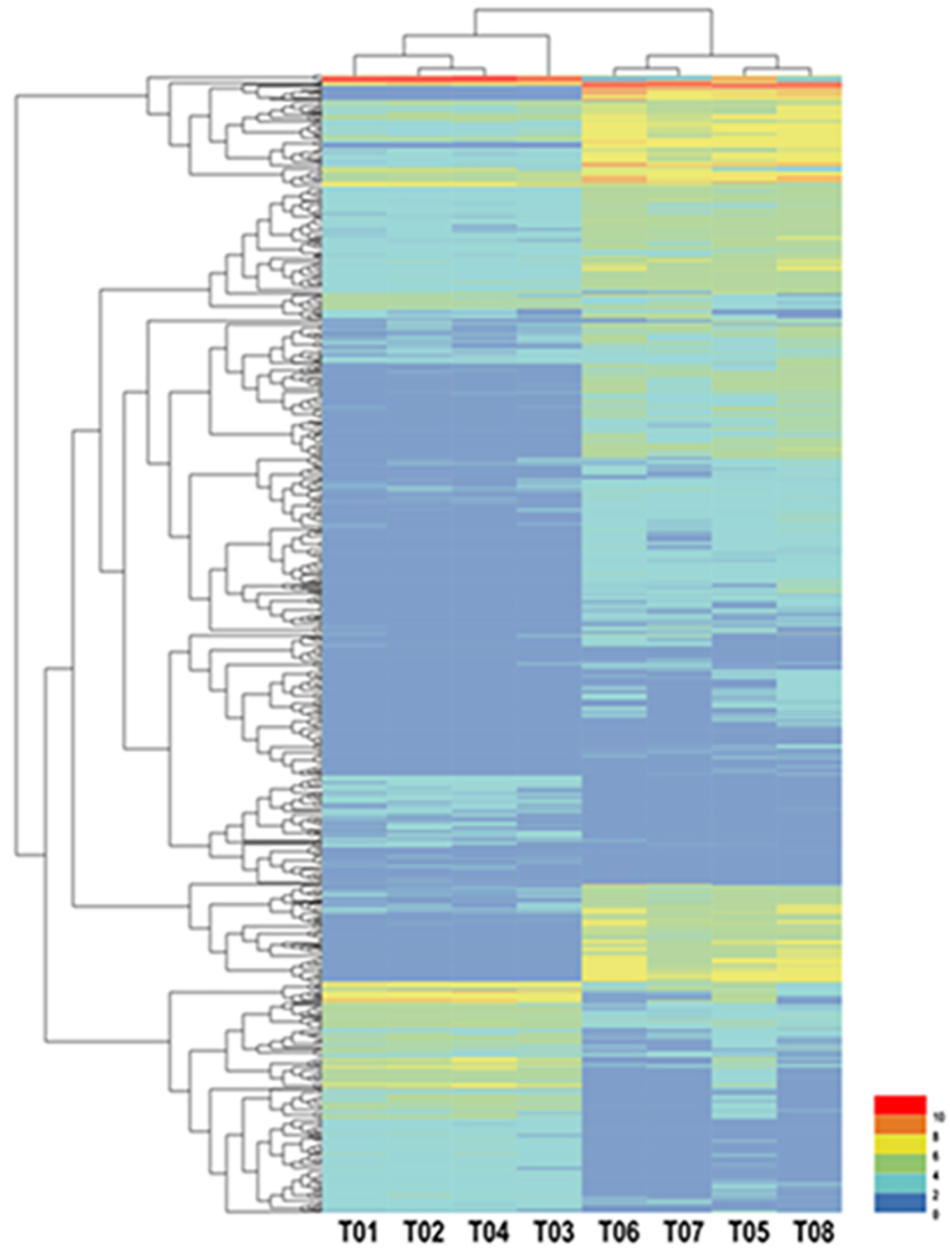
Hierarchical cluster analysis of blood clams (T01 T02 T03 T04 *versus* T05 T06 T07 T08). Each column represents a sample, each row represents a gene, and each different color represents log_2_ (Fragments per kilobase of transcript per million mapped reads (FPKM)) to indicate different expression levels. The clustering branch indicates similarity between genes or samples.

Several groups (T05 T08 *versus* T01 T02 T04, T06 T07 *versus* T01 T02 T04) were constructed to analyze DEGs using an FDR<0.01 and a |log2FC|≥1.The former group (T05 T08 *versus* T01 T02 T04) was identified to have 2,140 DEGs, containing 516 up-regulated and 1,624 down-regulated genes, while the latter (T06 T07 *versus* T01 T02 T04) had 2,070 DEGs, of which 795 were up-regulated and 1,275 were down-regulated. The relationship between the two groups is shown in Fig 2. In this study, we used the former group for further research, and created a volcano plot, shown in Fig 3. The volcano plot showed that male-biased DEGs were much more prevalent than female-biased DEGs, and that the expression level of male-biased DEGs varied significantly but were very stable in females. Hierarchical cluster analysis showed that the clustering branch displayed the similarity of genes or samples, which conformed to the evaluation of biological replicates (Fig 4).

#### DEG functional annotation (T05 T08 versus T01 T02 T04)

DEGs were aligned to several public databases to obtain functional annotations (E-value≤1×10^−5^). Consequently, 1346, 910, 971, 726, 307, 332 and 365 DEGs were annotated to Nr, Swissprot, PFAM, KOG, KEGG, GO, and COG databases, respectively.

#### Analysis of COG annotation

**Fig 5:**
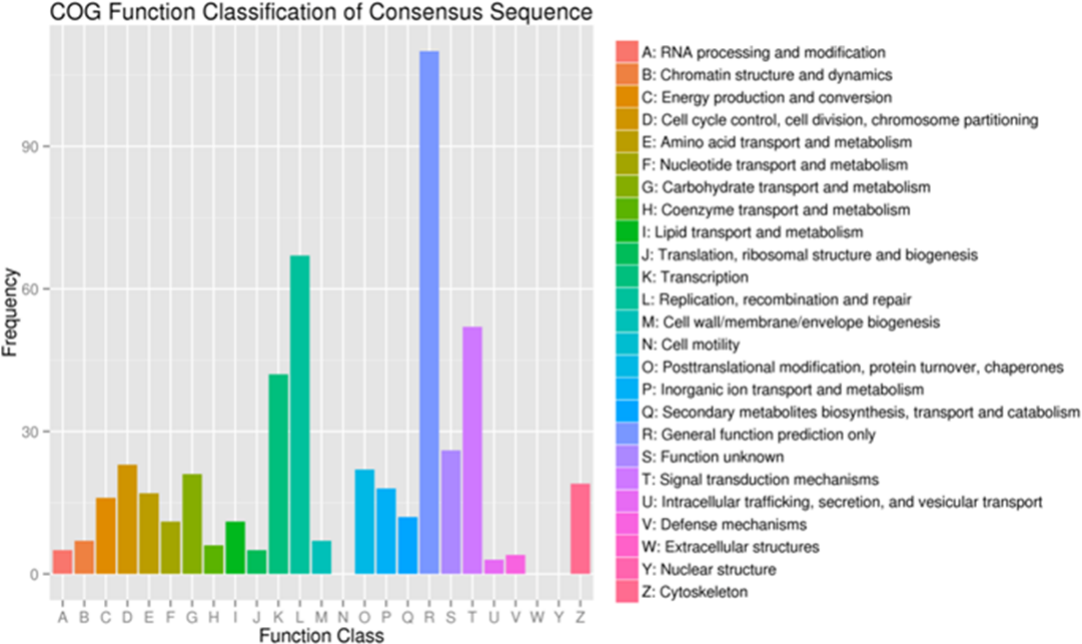
Cluster of Orthologous Groups (COG) functional classification of differentially expressed genes (DEGs) in blood clam (T05 T08 *versus* T01 T02 T04). The x-axis shows 25 categories while the y-axis shows the number of DEGs corresponding to each category.

COG, constructed by comparisons from a large number of prokaryotic protein sequences, is an early database for the identification and classification of orthologs. A total of 365 COG annotated DEGs were classified into 25 categories, among which general function prediction only (30.14%) was in the majority, followed by replication, recombination and repair (18.36%), signal transduction mechanisms (14.25%) and transcription (11.51%). COG annotation is shown in Fig 5.

#### Analysis of GO annotation

**Fig 6:**
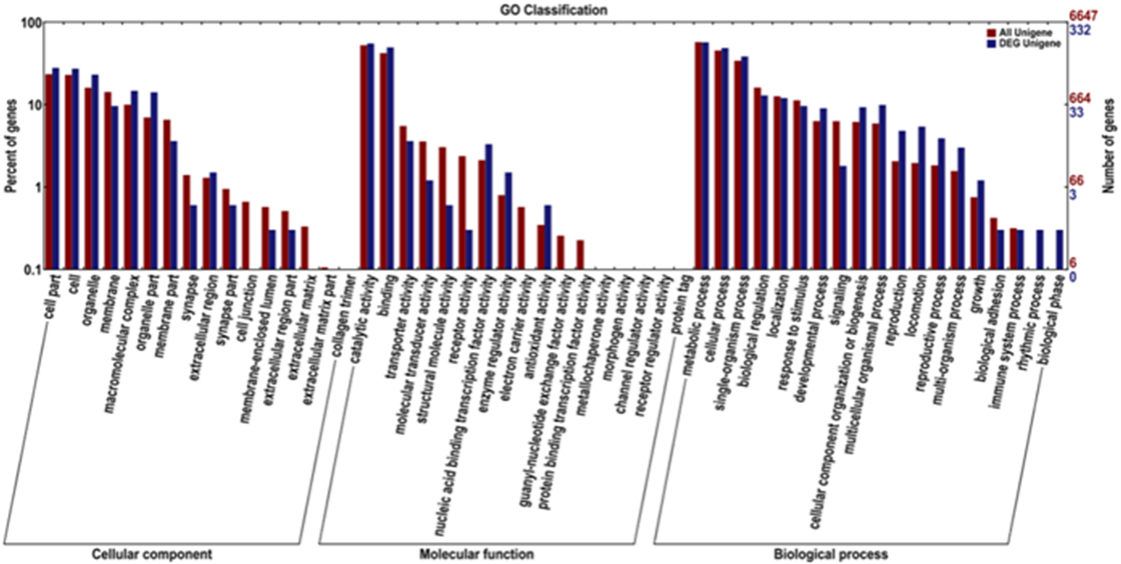
Gene Ontology (GO) functional classification of differentially expressed genes (DEGs) in blood clam (T05 T08 *versus* T01 T02 T04). The x-axis shows three terms and 52 sub-terms while the y-axis shows the proportion of DEGs and unigenes corresponding to each subcategory. The red column represents annotation of all genes, while the blue column represents annotation of DEGs. Under the background of the total genes and DEGs, a term having a large number of DEGs may be related to sexual differences.

GO is an international standardized database to describe features and functions of gene products. A total of 332 DEGs were assigned to 16 cellular components, 17 molecular functions and 19 biological processes. In the cellular component, most of the functions of DEGs were focused within the cell part (28.01%), cell (27.41%), and organelle (23.19%). In terms of molecular function, catalytic activity (55.42%) and binding (49.70%) contributed the largest proportion. With regard to biological processes, metabolic processes (57.23%) and cellular processes (48.80%) represented were the most prevalent. GO annotation is shown in Fig 6.

#### Analysis of KEGG annotation

**Fig 7:**
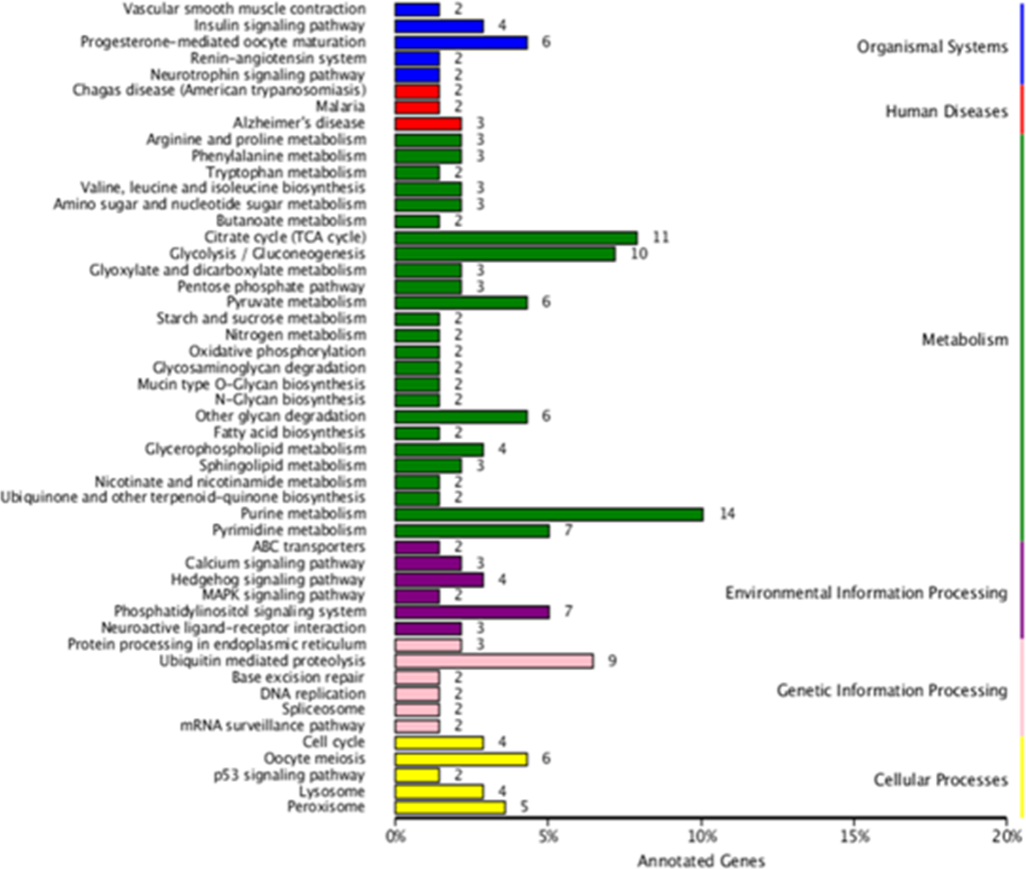
Partial Kyoto Encyclopedia of Genes and Genomes (KEGG) pathway of differentially expressed genes (DEGs) (T05 T08 *versus* T01 T02 T04). The x-axis shows 50 out of 104 pathways which contain more than one DEG while the y-axis shows the proportion of DEGs corresponding to each pathway.

KEGG is a database used to analyze the metabolic pathways and function of gene products, which integrates genomics, along with chemical, molecular and biochemical systems. A total of 307 DEGs were noted from KEGG. Of these, 139 DEGs were assigned to six categories, including organismal systems, human disease, metabolism, environmental information processing, genetic information processing and cellular processes. These were then mapped onto 104 pathways including purine metabolism (10.07%), citrate cycle (7.91%) and glycolysis/gluconeogenesis (7.19%), which represented the top three pathways (Fig 7). These data indicated that metabolic pathways play an important role in sex differentiation.

### Quantitative real-time PCR validation

As depicted in Fig 2, both of the two groups owned 1,327 DEGs when an FDR<0.001 and a |log2FC|≥1 were set as screening conditions. In this study, 35 DEGs and five sex-related unigenes in other similar species were investigated. Of the 40 unigenes, we were able to amplify all except for three down-regulated testicular unigenes. Of the 16 male-biased unigenes, M10 was a testis-specific gene while the other 15 genes were down-regulated with a fold change ranging from 3.825 to 40,322.961. Of the 16 female-biased unigenes, F4, F5, F10 were ovary-specific genes while the other 13 were up-regulated genes with a fold change varying from 3.310 to 177.465. Additionally, five sex-related unigenes were tested; in these, the fold change varied between 1.412 and 4.045, except for A5 (29.468) (Table 3). In brief, the qRT-PCR results substantially conformed to those of transcriptome sequencing, except for a few genes which were expressed at levels lower than expected, which may be related to the stability of *β-actin* and the amplification efficiency of our primers.

**Table 3:** qRT-PCR validation of transcriptome sequencing.

Functional classification	ID	Function	RNA-seq	qRT-PCR
Transcription	M1	Sox-14	166.664	170.386
	M2	Armadillo repeat-containing protein 4	11.295	53.269
	M3	Armadillo repeat-containing protein 3	6.678	14.705
	M4	Sox-8	533.812	2987.417
	M5	forkhead box J1 protein, partial	79.224	3.825
	F1	Foxl2	129.687	146.537
	F2	Forkhead box protein N2	115.624	133.897
	F3	Forkhead box E protein, partial	47.888	117.538
	F4	Spermatogenesis- and oogenesis -specific protein 2	23.519	—
	A1 (M)	Sox2	5.920	1.412
	A2 (F)	β-catenin	2.203	4.045
	A3 (F)	Dax1	1.344	1.766
	A4 (M)	Sox 9	1.593	2.174
	A5 (M)	DMRTA2	2.468	29.468
Signal transduction mechanisms	M6	Testis-specific serine/threonine-protein kinase 1	2264.369	1482.427
	M7	Troponin C, skeletal muscle	411.277	5803.698
	M8	Testis-specific serine/threonine-protein kinase 1	1939.053	1056.228
	M9	Sperm motility kinase X	334.901	3955.033
	M10	Testis-specific serine/threonine-protein kinase 5	280.606	—
	M11	Testis-specific serine/threonine-protein kinase 4	1832.153	4594.291
Carbohydrate, Lipid, Amino acid transport and metabolism	M12	Glycogen phosphorylase, muscle form	1523.181	40322.961
	M13	Tax1-binding protein 1-like protein B	163.139	73.838
	F5	Vitellogenin-6	2090.712	—
	F6	Chymotrypsin-like elastase family member 2A	17.829	2.962
	F7	Chymotrypsin-like serine proteinase	17.423	7.342
	F8	Chymotrypsin-like elastase family member 2A	14.892	4.283
	F9	Chymotrypsin-like serine proteinase	16.022	5.073
Egg coated protein	F10	vitelline envelope zona pellucida domain 4	129.305	—
	F11	vitelline envelope zona pellucida domain 10	12.964	7.139
	F12	vitelline envelope zona pellucida domain 10	597.534	177.465
	F13	vitelline envelope zona pellucida domain 10	12.382	3.310
Immune-related protein	M14	Sperm-associated antigen 6	20.916	41.411
	F14	placenta-specific gene 8 protein-like	20.263	31.953
	F15	Placental protein 11	7.582	10.440
Cell cycle control	M15	F-box only protein 39	350.894	6.619
	F16	G2/mitotic-specific cyclin-B	193.215	159.333
Chromatin structure and dynamics	M16	Sperm-specific protein PHI-2B/PHI-3	716.255	2678.482

‘–’ represents male or female-specific genes, M represents males, F represents females and fold change indicates the differential change in expression between the two genders.

### Sex determination and differentiation genes

We carried out a search of the known sex determination/differentiation genes in animals, focusing mostly on *Mus musculus* (mouse), *Danio rerio* (fish), *Drosophila melanogaster* (fly), *Caenorhabditis elegans* (worm) and *Crassostrea hongkongensis* (oyster) (Table 4). In total, 23 out of 43 genes were detected in blood clam transcriptome, and were similar to those of *C*. *hongkongensis*[48]. Of these 23 genes, *CBX8* and *Foxl2* were female-biased, while the family of *Sox*, *armadillo-catenin* and *Sxl* were male-biased. Other common sex determination genes, such as *ATRX*, *DMRT*, *Dax1*, *Wnt4*, *Tra-2*, and *Fem-1*, showed no significant difference between males and females and appeared to express at the highest level during early stages of development and recover to normal levels as animals matured.

**Table 4:** Candidate genes for sex determination and differentiation in model organism.

	Gene source	*C*.*hongkongensis*	*T*.*granosa*(homologues)
*WT1*	Mouse		
*Sf1*	Mouse		
*LHX9*	Mouse	Y	*LHX9*
*EMX2*	Mouse		*EMX1*
*GATA4*	Mouse	Y	*GATA4*
*SRY*	Mouse		
*Sox*	Mouse	Y	Sox2*(M), Sox8**(M), *Sox9* Sox14**(M)
*RSP01*	Mouse		
*FOG2*	Mouse		
*AMH*	Mouse		
*DMRT*	Mouse		*DMRTA2*
*MAP3K*	Mouse	Y	*MAP3K1*, *MAP3K4*
*ATRX*	Mouse	Y	*ATRX*
*FGF9*	Mouse		*FGF18*
*Gadd45g*	Mouse	Y	*Gadd45g*
*Hhat*	Mouse	Y	
*Kdm3a*	Mouse		
*Dax1*	Mouse	Y	*Dax1*
*Six*	Mouse	Y	*Six1*, *Six3*, *Six4*
*GSDF*	Mouse		
*PDGF*	Mouse		
*AR*	Mouse		
*SRD5A1*	Mouse	Y	
*WNT4*	Mouse	Y	*Wnt4*
*FOXL2*	Mouse	Y	Foxl2**(F)
*β-catenin*	Mouse	Y	*Armadillo/beta-catenin* Armadillo/beta-catenin**(M)
*FST*	Mouse	Y	*FST*-like
*cyp11b*	Fish		
*cyp19A1*	Fish		
*ERa*	Fish	Y	*ERa*
*Fhl2*	Fish		*Fhl2*
*Ixl*	Fish		
*Xol-1*	Worm		
*Sdc*	Worm		
*Her*	Worm		
*Tra*	Worm	Y	*Tra-2*
*Fem*	Worm	Y	*Fem-1*
*CBX*	Fly		*CBX5*, CBX8**(F)
*Fru*	Fly		
*Sis*	Fly		
*Runt*	Fly	Y	*Runt*
*Sxl*	Fly		Sxl**(M)
*Doa*	Fly	Y	*Doa*

Y represents the presence of these genes or their homologues in *C*. *hongkongensis* while the fourth column represents the homologues of these genes in *T*. *granosa*. M indicates significant expression in males while F indicates significant expression in females.

* indicates a significant difference (*P*<0.05) while

** indicates a highly significant difference (*P*<0.01).

### SSRs and SNPs

**Fig 8:**
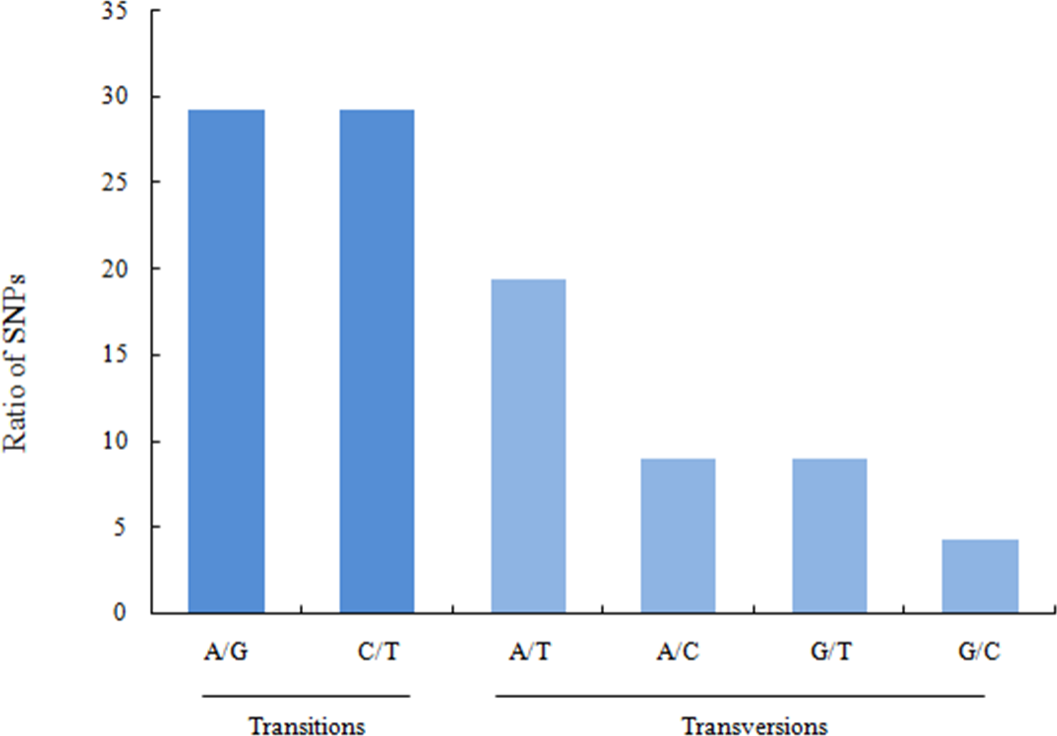
Singlenucleotidepolymorphism (SNP) types showing polymorphism of the sexual transcriptome sequence of the blood clam.

Structural analysis of unigenes detected 6,283 SSR markers, of which di -nucleotide was the most predominant (62.41%), followed by tri- (21.36%), tetra- (2.05%), penta- (0.10%), and hexa-nucleotides (0.03%) (Table 5). In addition, 614,710 SNPs were also detected, including 58.53% transitions (A/G 29.30% and T/C 29.23%) and 41.47% transversions (A/T 19.33%, A/C 8.93%, G/T 8.92%, G/C 4.30%) (Fig 8).

**Table 5:** Types of simple sequence repeats (SSR) identified in the gonadal transcriptome of the blood clam.

Repeat motif	Number	Percentage (%)
Di-nucleotide		
AC/CA/GT/TG	347/348/424/378	
AG/GA/CT/TC	200/270/119/162	
AT/TA/GC/CG	872/800/0/1	
Total	3921	62.41%
Tri-nucleotide		
AAC/AAG/AAT(N≥5)	43/16/139	
ACA/ACC/ACG/ACT	40/22/3/2	
AGA/AGC/AGG/AGT	13/1/3/5	
ATA/ATC/ATG/ATT	95/34/48/88	
CAA/CAC/CAG/CAT	46/10/8/33	
CCA/CCT/CGC	16/4/1	
CTA/CTC/CTG/CTT	4/6/6/6	
GAA/GAC/GAG/GAT	20/3/6/48	
GCA/GCT/GGA/GGT	10/6/3/11	
GTA/GTC/GTG/GTT	3/2/12/18	
TAA/TAC/TAG/TAT	63/9/3/81	
TCA/TCC/TCG/TCT	52/4/3/14	
TGA/TGC/TGG/TGT	66/7/11/36	
TTA/TTC/TTG	108/15/36	
Total	1342	21.36%
Tetra-nucleotide		
AAAC/AAAT	3/9	
AACA/AATA/AATC/AATT	3/10/5/1	
ACAG/ACAT/ACGC/ACTG	1/2/1/1	
AGAA/AGAT/AGTG	1/1/1	
ATAA/ATAC/ATAG/ATGT	5/3/2/3	
ATTA/ATTG/ATTT	1/2/8	
CAAA/CAAC/CTAT/CTGT	1/2/1/1	
GAAA/GAAT/GACA/GATA	1/2/1/1	
GTAT/GTCA/GTCC/GTCT	1/1/1/2	
GTGC/GTTG/GTTT	1/1/1	
TAAA/TAAT/TACA/TACT	4/1/1/1	
TATC/TATG/TATT	3/2/6	
TCAA/TCAT/TCTT	1/2/1	
TGAC/TGTA/TGTC	1/1/2	
TTAA/TTAT/TTGA/TTGT	1/7/1/1	
TTTA/TTTC/TTTG	7/2/3	
Total	129	2.05%
Penta-nucleotide		
AAAAT/AATCC/TGAGT	1/1/1	
CAAAG/CAGGC/CCAGC	1/1/1	
Total	6	0.10%
Hexa-nucleotide		
TTTTTC/TTATAA	1/1	
Total	2	0.03%
others	883	14.05%

‘Number’ indicates the number of different types of SSR detected in unigenes while‘Percentage’ indicates the relative proportion of SSRs with different repeat motifs among the total number of SSRs.

## Discussion

Blood clams are one of the most economically important marine bivalves. In recent years, the sex ratio of this shellfish has become distorted and female fecundity has decreased, resulting in gamete deformity and a decline in yield. Consequently, elucidating the reproductive biology of shellfish is important in understanding embryonic and individual development, reproduction and population structure. In addition, the sex differentiation and sex regulation mechanisms of bivalves vary significantly, and existing studies on sex-related genes remain insufficient. Therefore, it was necessary to construct a sexual transcriptome for this important species.

### Elucidation of the blood clam transcriptome

To build up a gonadal expression profile from the blood clam, 6G of each sample was sequenced using an Illumina HiSeq2500 high-throughput sequencing platform; this identified a total of 6,772,406 contigs, 214,440 transcripts and 125,673 unigenes. Furthermore, 6,283 SSRs and 614,710 SNPs were also determined. Compared with other bivalves, this was the largest gonadal transcriptome data so far; For the blood clam, there was only one existing resource, a microRNA transcriptome associated with resistance response to Cd^2+^; the present study identified the first transcriptome relating to gonadal expression profile in blood clams, which will provide a useful resource for the future study of mechanisms underlying the function of sex-related genes in bivalves.

### DEGs analysis of males and females

DEGs were allocated to 104 pathways by KEGG; the most dominant pathways related to purine metabolism, followed by the citrate cycle and glycolysis/gluconeogenesis. Purine can be converted into ATP which is used for the storage and supply of energy while purines may also be transformed into cAMP and used as a second messenger to regulate metabolism and physiological activities. Glycolysis/gluconeogenesis are processes used to metabolize glycogen into pyruvate and thus produce energy. The DEGs identified in our study which related to these three processes were predominantly male-biased. Therefore, we may draw the conclusion that energy metabolism in males may be higher than females.

The validated DEGs are functionally classified into 7 categories, including Transcription, Signal transduction mechanisms, Carbohydrate, lipid, amino acid transport and metabolism, Egg coated protein, Immune-related protein, Cell cycle control and Chromatin structure and dynamics. Transcription factors serve as sex determination genes which determine the direction of gender differentiation. Genes annotated in signal transduction pathway mostly are kinase with the ability to transfer the different gender development signals intercellularly. Genes annotated in signal transduction pathway mostly are kinase with the ability to transfer the different gender development signals intercellularly. The transport and metabolism genes of Carbohydrate, lipids and amino acids, three important metabolites, are essential for hormone synthesis. Moreover, the egg coated protein and immune-related protein, the cell cycle genes, the chromatin structure and dynamics genes play an irreplaceable roles in protection of gametes and organs, the controlling of cell apoptosis process, and the compression of chromatin, respectively.

### Candidate sex-related genes

*Sox* is a family of transcription factors with a high mobility group (HMG) domain which can bind and bend DNA. The *Sox* family has more than 20 homologues ranging across different species. In vertebrates, *Sry* is the main promoter of sexual differentiation and functions only in mammals. *Sox9* is the only known target of *Sry* and over-expression of *Sox9* can substitute for the function of *Sry* during testis determination. *Sox8* can reinforce *Sox9* function during testis differentiation and can even replace *Sox9* when *Sox9* is either not expressed or is expressed too late. Moreover, *Sox8* and *Sox9* are critical for the maintenance of male fertility[14–15]. Other members of the *Sox* family, such as *Sox5*, *Sox6*, and *Sox13* also play roles in spermatogenesis. In invertebrates, we were able to identify *Sox100B* (ID: 45039) in *Drosophila melanogaster* and *Sox8* (*SoxE*, ID: 105340517) in *Crassostrea gigas* from NCBI. In our present study, we identified *Sox2*, *Sox8*, *Sox9*, and *Sox14*, and showed, with the exception of *Sox9*, that these genes have a significant difference in gene expression when compared between males and females. Although the detailed function of *Sox9* is unknown, we speculate that this gene may function during earlier stages, and that *Sox8* is more likely to replace the function of *Sry* in the determination of testes in the blood clam.

*Foxl2* is a member of the fork head (FKH) family with a winged helix domain, which was originally identified in *Drosophila*. *Foxl2* functions as an important transcription factor which is indispensable for ovarian development and the growth and maturation of ovarian follicles[31]. In vertebrates, *Foxl2* localizes to the granulosa cells and the early ovarian stroma, and knockout of this gene triggers disorder in ovarian follicular formation and partial ovary-to-testis sex reversal. In addition, *Foxl2* can upregulate the expression of the *P450* aromatase gene which converts androstenedione and testosterone into estrone and estradiol[68], and acts for extended periods of time throughout ovarian development. Homologues of *Foxl2* have also been reported in invertebrates[43,69,70]. We also detected *Foxl2* in the transcriptome of blood clam. *Foxn2* and *Foxe*, belong to the FKH family and show significant differences among individuals from the two genders, suggesting that these three genes may be involved in ovarian determination, although the precise mechanisms involved remain uncertain.

*β-catenin* plays a key role in the Rspo1/Wnt signaling pathway which has been associated with ovarian determination. *β-catenin* has three components: an N-terminal used for GSK-3*β* phosphorylation, a central region consisting of 12 armadillo (ARM) repeats, and a C-terminal which has a transactivation domain. In mice, the activation of *β-catenin* by *Wnt4* and *Rspo1* effectively blocks the testis pathway, leading to male-to-female sex-reversal. Moreover, *β-catenin* is antagonistic to *Sox9*, resulting in differentiation towards the female pathway[29]. In mollusks, the Armadillo repeat region is a *β-catenin* ortholog and has been reported in *C*.*gigas*, *C*.*hongkongensis* and *C*. *farreri*[48, 71–72]. *β-catenin* is expressed at much higher levels in mature female gonads than those in male gonads. However, many Armadillo repeats are found in the blood clam transcriptome, some of which show no significant difference between males and females, while others are expressed at higher levels in males than in females. Of the genes containing Armadillo repeats, there are many genes recongised as sperm-associated antigens, suggesting that Armadillo repeats may play an important role in spermatogenesis. Therefore, we speculate that Armadillo repeats are more essential in male blood clams compared to females.

*CBX* (chromobox homolog) genes are members of the PcG family, which are major epigenetic regulators. *CBX8* has been described as an epigenetic transcriptional repressor involved in the inhibition of cell senescence, proliferation and metastasis of cancer cells[73–74] while *CBX7* is involved in the modulation of cell apoptosis and gene transcription in several cell types[75]. Beyond this, *CBX2*/*M33* functions as a critical factor in controlling the meiotic process of male germ cells[76]. *CBX2* is a female-biased gene in the mouse, and the targeted ablation of this gene leads to male-to-female sex reversal[77]. However, *CBX2* also functions during testis differentiation by regulating genetic expression of *Sry*[78]. In the present study, we demonstrated *CBX5* and *CBX8* to be significantly expressed in the female blood clam. We therefore hypothesize that these two genes are regulators of the developmental process of germ cells, although the specific mechanisms involved remain unknown.

The *Sxl* gene in *Drosophila melanogaster* encodes an RNA-binding protein, which controls the regulation of sex determination pathways. In brief, gender in *D*. *melanogaster* is determined by the X:A signal; double doses of X in females initiates *Sxl* expression, and the *Sxl* protein regulates the splicing of *Tra*^F^mRNAs into a female-specific form. In addition, the *Sxl* protein also establishes an auto-regulatory feedback loop to dominate the splicing of *Sxl*^F^mRNAs, thus maintaining sex stability throughout development. In contrast, a single dose of X in males cannot activate *Sxl* protein expression, and the subsequent regulatory strategy will be different[41]. In the blood clam transcriptome, a *Sxl*-like gene was significantly expressed in males, which is contrary to that of *D*. *melanogaster*. We speculate that the *Sxl* regulatory strategy is perhaps not applicable to *T*. *granosa* which has no sex chromosomes, and the precise meaning of this key difference is still being investigated.

Our transcriptome is the most complete database for blood clam thus far. We not only identified many homologues of sex-related genes which have been reported in other species, but also found many other unigenes in our current transcriptome which related to our specific commercial needs, such as genes associated with immunity and growth. Our new database provides a relatively complete gene sequence for further analysis and represents a firm foundation for a range of further research studies.

## Conclusions

In conclusion, our study provided the first gonadal transcriptome data for blood clam, a commercially important shellfish along the southeast coastline of China. We used non-reference transcriptional sequencing due to the insufficient amount of genetic information available for this species. Based upon COG, GO and KEGG classifications, we were able to elucidate the function of DEGs in specific pathways. This data will be beneficial in improving our understanding of the transcriptomics of blood clams, while SSRs and SNPs will be useful for genetic evolution analysis, bulked segregant analysis (BSA) and genome-wide association studies (GWAS), thus providing a theoretical basis for genetic breeding and the conservation of germplasm.
